# Mutations at codons 178, 200-129, and 232 contributed to the inherited prion diseases in Korean patients

**DOI:** 10.1186/1471-2334-9-132

**Published:** 2009-08-22

**Authors:** Bo-Yeong Choi, Su Yeon Kim, So-Young Seo, Seong Soo A An, SangYun Kim, Sang-Eun Park, Seung-Han Lee, Yun-Ju Choi, Sang-Jin Kim, Chi-Kyeong Kim, Jun-Sun Park, Young-Ran Ju

**Affiliations:** 1 Division of Arboviruses, Center for Immunology and Pathology, National Institute of Health, Korea Centers for Disease Control and Prevention, Seoul, Republic of Korea; 2 Department of Bionanotechnology, Kyungwon University Gachon Bionano Research Institute, Seongnam, Republic of Korea; 3 Department of Neurology, Seoul National University College of Medicine, Seongnam, Republic of Korea; 4 Department of Neurology, Seoul Veterans Hospital, Seoul, Republic of Korea; 5 Department of Neurology, Chonnam National University Medical School, Gwangju, Republic of Korea; 6 Department of Neurology, Busan-Paik Hospital, Inje University College of medicine, Busan, Republic of Korea

## Abstract

**Background:**

Polymorphisms of the human prion protein gene (*PRNP*) contribute to the genetic determinants of Creutzfeldt-Jakob disease (CJD). Numerous polymorphisms in the promoter regions as well as the open reading frame of *PRNP *were investigated. Greater than 90% of Korean, Chinese, and Japanese carry the homozygote 129 MM codon. In Korea, polymorphisms have not been comprehensively studied, except codons 129 and 219 in *PRNP *among Korean CJD cases. Although polymorphisms at codons 129 and 219 play an important role in susceptibility to sporadic CJD, patients with other polymorphisms in *PRNP *exhibited critical distinctions of clinical symptoms.

**Methods:**

The genetic analyses of *PRNP *were carried out among probable CJD patients in comparison with the results from magnetic resonance imaging (MRI) and electroencephalogram (EEG).

**Results:**

The molecular analyses revealed that three mutations at codons D178N, E200K, and M232R in heterozygosity. Patients with the D178N and M232R mutations had a 129MM codon, whereas the patient with the E200K mutation showed 129MV heterozygosity. They all revealed strong 14-3-3 positive signals. The 67-year-old patient with the D178N-129M mutation showed progressive gait disturbance and dysarthria was in progress. The 58-year-old patient with the E200K mutation coupled to the 129MV codon had gait disturbance, dysarthria, agitation, and ataxic gait, and progressed rapidly to death 3 months from the first onset of symptoms. The 65-year-old patient with the M232R mutation showed rapidly progressive memory decline and gait disturbance, and died within 16 months after onset of symptoms.

**Conclusion:**

Despite differences in ethnicity, the clinical and pathological outcomes were similar to the respective mutations around the world, except absence of insomnia in D178N-129M subject.

## Background

Creutzfeldt-Jakob disease (CJD), Gerstmann-Sträussler-Scheinker syndrome (GSS) fatal familial insomnia (FFI), and Kuru are transmissible spongiform encephalopathies (TSE) diseases in human. TSEs are typically fatal neurodegenerative diseases; 90% of CJD patients die within 1 year of diagnosis which occurs sporadically at an annual incidence of 1 per million populations [1,2]. Among all CJD cases, 10–15% has been reported as autosomal dominant disorders, with mutations in the prion protein gene (*PRNP*) on chromosome 20, and is categorized as genetic TSE. These reports have suggested the importance of *PRNP *mutations in familial CJD (fCJD). *PRNP *mutations have been discovered from more than 30 sites, and lead to amino-acid substitutions, premature stop codons, or the insertion of additional octapeptide repeats at the N-terminus [3]. Some could be transmitted in an autosomal dominant inheritance pattern, with nearly 100% penetrance [4,5]. These findings emphasize the importance of investigating *PRNP *polymorphisms or mutations to predict disease occurrence.

As a rapidly progressing neurodegenerative disorder, the symptoms of CJD are characterized by progressive dementia, ataxia, and myoclonus [6]. Familial CJD tends to have an earlier age of onset and longer duration than sporadic CJD (sCJD). The E200K mutation is the most common in fCJD (more than 70% worldwide) with the D178N mutation being the second most frequent [7]. The effect of codon 129 on the phenotype associated with the E200K mutation seems to be less distinguishable than for the D178N mutation [8]. Hence, the phenotypic effect of the D178N mutation depends on polymorphism at codon 129 of *PRNP*. Fatal familial insomnia (FFI) seems to be associated with a D178N mutation and methionine at codon 129 of *PRNP*, whereas the phenotype of sCJD was observed for the D178N mutation with valine at codon 129 [9]. The D178N mutation almost never indicated the characteristic electroencephalogram (EEG) changes, but this was not the case when it was linked with the E200K mutation [1].

The clinical, EEG and neuroimaging features in patients with E200K-129M mutation were similar to sCJD. A typical EEG, with periodic spike and wave (PSW) complexes, was observed in about 75% of all patients [10]. The levels of 14-3-3 protein in the cerebrospinal fluid (CSF) increased in almost all cases [11]. The phenotype of patients with E200K-129V mutations was similar to that of patients categorized as CJD VV2 type. The typical presenting symptom was ataxia followed by myoclonus and PSW complexes on EEG [12].

The M232R mutation was reported in eight Japanese patients without any previous family history of neurodegenerative diseases [13]. The clinical feature of M232R mutation was similar to that of sCJD. Common symptoms were progressive memory impairment, gait disturbance, and myoclonus, and a typical EEG with PSW complexes was observed in all cases except in an 84-year-old subject [14].

CJD is clinically diagnosed with specific finding with magnetic resonance imaging (MRI), periodic sharp and wave complexes (PSWCs), and elevated 14-3-3 protein in the CSF [15-17]. The detection of 14-3-3 protein in CSF is an important marker supporting the diagnosis of CJD [16,18]. Although the positive detection of 14-3-3 protein has been reported in other neurological disorders [19], the diagnostic criteria of the World Health Organization (WHO) for CJD includes 14-3-3 detection [20].

In this study, the mutations of *PRNP *at codons 178, 200-129, and 232 reported in other countries were first discovered among probable CJD patients in Korea. In addition, the level of 14-3-3 protein released into the CSF was studied and compared with the MRI/EEG results.

## Methods

### Patient history

The clinical findings of probable CJD patients in Korea are summarized in Table 1.

**Table 1:** The clinical findings of probable CJD patients in Korea with codons 178, 200-129, and 232 mutations.

Patient	Case 1	Case 2	Case 3
Age of onset	67	58	65
Mutation	D178N-129M	E200K-129MV	M232R
Sex	M	M	M
Symptoms of onset	Progressive gait disturbance Dysarthria Extrapyramidal sign	Gait disturbance Confused mentality Dysarthria Agitation Myoclonus	Memory decline Gait disturbance
Total clinical duration	Alive (27 months)*	3 months	16 months
MRI imaging (DWI or FLAIR)	High signal intensities in both parietal and occipital gyri	High signal intensity on bilateral frontotemporoparietal area and caudate nucleus	High signal intensities from the cortex of the parieto-occipital and the temporal lobes
EEG finding	Normal	Sharp or spike and slow waves	Diffuse theta to delta range slow waves
14-3-3 protein in CSF	Positive	Positive	Positive

* As of May 2008

#### Case 1

A 67-year-old man was admitted to hospital because of progressive gait disturbance and dysarthria. He didn't have family history of similar problems. The subject also had extrapyramidal symptoms such as rigidity and bradykinesia, but no sign was evident of myoclonus, visual symptoms, pyramidal symptoms, or cognitive impairment. The cerebellar function test was conducted by tandem gait, heel-to-shin, finger-to-nose, and rapid alternating movements, and the results were normal. The laboratory findings for electrolytes, antibodies of the thyroid gland, liver function test, immunological test for syphilis, and viral markers were negative. His CSF was clear and colorless without any white blood cells. A few months after onset the patient's health deteriorated gradually into akinetic mutism, which progressed further.

#### Case 2

A 58-year-old man was hospitalized with gait disturbance and confused mentality, and there was no family history. He had fallen from a bike 2 weeks prior to his visit to hospital. He had stumbled, could not walk in a straight line, and frequently fell down. He presented with a history of chronic alcoholism and hypertension. His symptoms progressed and his condition deteriorated, with dysarthria and agitation. He could not perform the high cortical function test due to reduced attention, and he developed an ataxic gait. Although he looked cautious during the neuropsychological test, he showed frequent disorientation and confusion, and often fell into a stupor. He was not able to be woken from sleep on the 6th day after hospitalization, and on the 12th day developed aspiration pneumonia, which was treated. He later developed myoclonic movements in his limbs. The laboratory findings for electrolytes, antibodies of the thyroid gland, liver function test, immunological test for syphilis, and viral markers were negative. His CSF was clear and colorless without any white blood cells. He died 3 months after the first onset of neurological signs of CJD.

#### Case 3

A 65-year-old man presented with rapidly progressive memory decline and gait disturbance. He had no known diabetes, cardiac disease, or previous stroke except hypertension. Also, he had no family history of dementia or overseas travel, he was alert. Cranial nerve, sensory, reflex, cerebellar function, and motor power examinations did not revealed any abnormality. But he showed a bilateral swaying gait. The cognitive function test showed frontal lobe dysfunction, and his mini-mental state examination score was 22. His rating score for clinical dementia was 0.5, and his Barthel index was 14. The laboratory findings for electrolytes, antibodies of the thyroid gland, liver function test, immunological test for syphilis, and viral markers were negative. His CSF was clear and colorless without any white blood cells. His symptom progressed further and he died within 16 months after onset of symptoms.

### Samples

Blood and CSF samples were collected from 99 probable CJD patients in Korea. Genetic analyses of their *PRNP *sequences revealed mutations at D178N-129M, E200K-129MV, M232R, 129MV, E219K, E219K-129MV, P102L, and V180I. Among them, further analyses were carried out on three patients with mutations at D178N-129M, E200K-129MV, and M232R under their consents for research only. Control DNA was obtained for analysis from healthy volunteers. This study was approved by the Institutional Review Board (IRB).

### Western blotting

A recombinant 14-3-3β plasmid cloned into the expression vector pEF6/V5-His TOPO was transiently transfected into 293T cells using FuGENE 6 (Roche Applied Sciences, Basel, Switzerland). The CSF (32 μl) from probable CJD patients were separated on a 4–12% Bis-Tris gel, then transferred to a PVDF (polyvinylidene fluoride) membrane, followed by 5% dry milk. Polyclonal antibodies specific to 14-3-3β (Santa Cruz Biotechnology, sc-629) were used for blotting. The signals were developed using a SuperSignal^® ^West Pico Chemiluminescent Substrate (PIERCE, IL, USA). CSF from definite sCJD patient was used as the positive control.

### DNA preparation and sequence analysis

Genomic DNA of probable CJD patients was extracted from 200 μl blood samples using the QIAamp DNA Blood Mini Kit (Qiagen), following the manufacturer's protocol. Polymerase chain reaction (PCR) was performed with the S13 (5'-AAGCCTGGAGGATGGAACAC-3') and T2 (5'-CCCACTATCAGGAAGATGAG-3') primers, which amplified nucleotide 79–758 of *PRNP*. The 30 cycles of PCR were performed as follows: 94°C for 1 min, 60°C for 45 sec, and 72°C for 1 min 30 sec. PCR products for sequencing were purified using a Gel Extraction Kit (Qiagen, MD, USA). DNA sequencing with primers, S13 and T2, was carried out using a BigDye^® ^Terminator v3.1 Cycle Sequencing Kit (Applied Biosystems) on an ABI PRISM 3730xl DNA Analyzer (Applied Biosystems, CA, USA). Results of DNA sequencing were analyzed using Sequencing Analysis Software (Applied Biosystems, CA, USA).

### SNaPshot (Single Nucleotide Primer Extension) assay

SNaPshot was analyzed with the SNaPshot Multiplex Kit according to the ABI protocol, using PCR products amplified under the same conditions as for DNA sequencing (Applied Biosystems, CA, USA). SNaPshot primers were S178 (5'-TTGATTGTGATATTGACGCAGT-3') for the codon 178 variants, S129 (5'-GTGGTGGGGGGCCTTGGCGGCTAC-3') for the codon 129 variants, S200 (5'-AAAAAAAAAACCACCAAGGGGGAGAACTTCACC-3') for the codon 200 variants, and S232 (5'-TATTACCAGAGAGGATCGAGCA-3') for the codon 232 variants. The samples were analyzed on an ABI PRISM^® ^3100 Genetic Analyzer (Applied Biosystems, CA, USA), and the data were analyzed with GeneScan software (Applied Biosystems, CA, USA).

### Routine restriction fragment length polymorphism (RFLP)

Each PCR products amplified using the same condition as for the DNA sequencing for Cases 1 and 2, were digested with the restriction enzymes Tth111 I, Nsp I, and BsmA I (New England Biolabs). The PCR conditions for Case 3 were as follows: 30 cycles of 94°C for 30 sec, 60°C for 1 min, and 72°C for 1 min with the K-7 (5'-GTCACCACAACCACCAAGGG-3') and K-8 (5'-CAGGAAGACCTTCCTCATCC-3') primers. The PCR products for Case 3 were then digested with Nla III (New England Biolabs, Hitchin, UK). The resulting fragments were separated by electrophoresis on a 10% TBE gel (Bio-Rad Ready Gel), and stained and visualized using EtBr.

## Results

### Detection of the 14-3-3 protein in CSF samples from probable CJD patients

**Figure 1:**
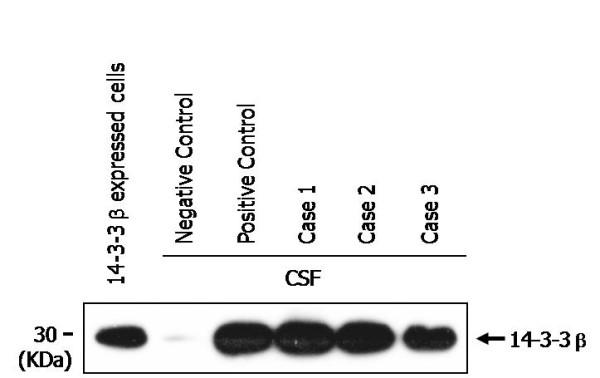
**Increased 14-3-3 was observed in CSF from probable CJD patients using immunoblot**. CSF from healthy control and probable CJD patients were compared with 293T cell transfected with the pEF6/V5-His plasmid encoding 14-3-3β, and the CSF of a definite sCJD patient as positive control.

In the Western blot of the 14-3-3 protein, band from the 293T cells transfected with 14-3-3β expression plasmid matched exactly the bands from the CSF of the positive control and probable CJD patients, confirming the bands from probable CJD patients as 14-3-3. The 14-3-3 appeared to have slight signal in the CSF of negative control, which was obtained from a patient with Japanese encephalitis virus (JEV). Later, this patient was diagnosed as normal. The detected levels of 14-3-3 in CSF from Cases 1, 2, and 3 were similar, although mutated codons in *PRNP *were different (Figure 1).

### DNA sequencing analysis and SNaPshot

**Figure 2:**
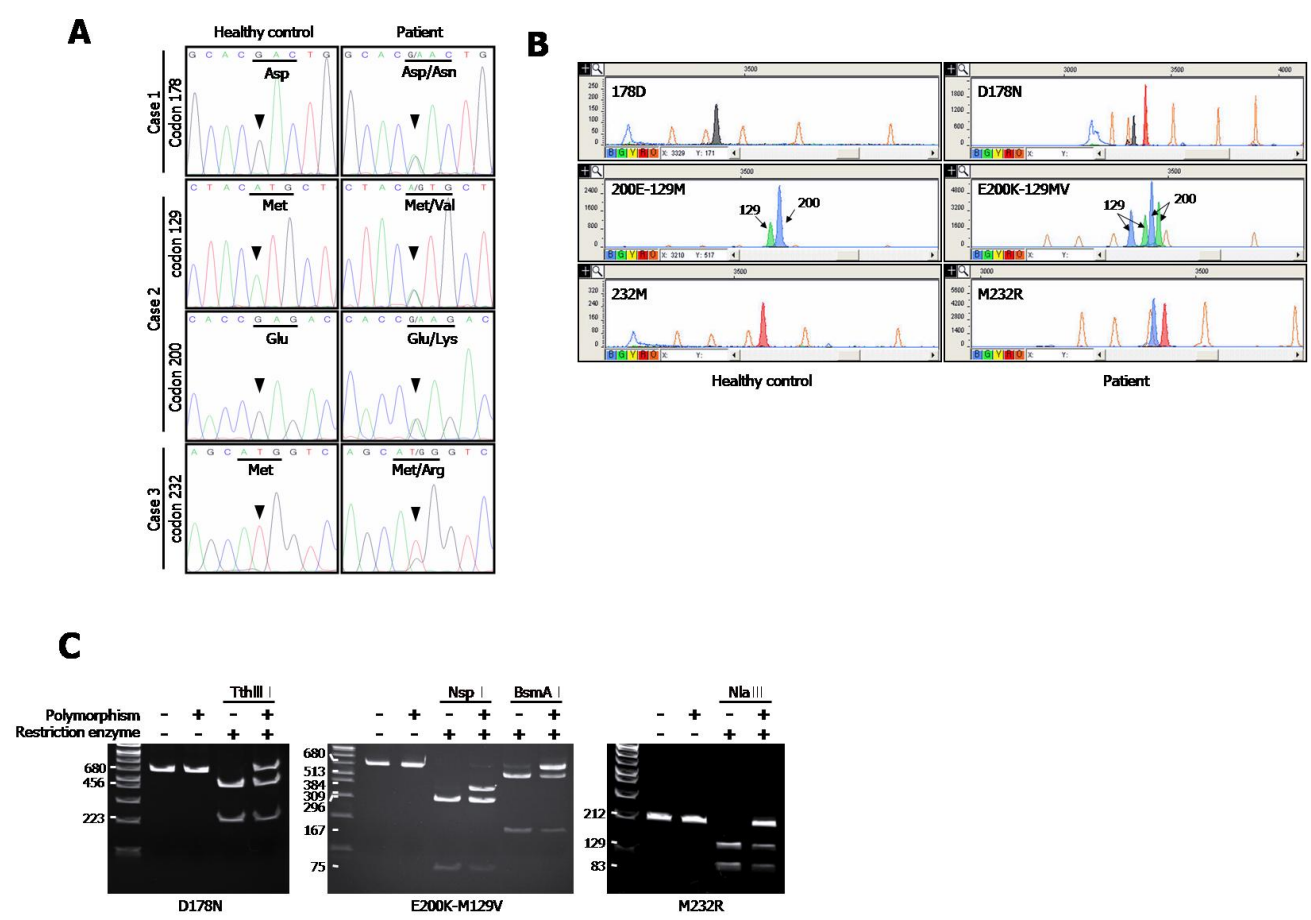
**Mutations in *PRNP *were detected from probable CJD patients**. Electropherogram of the DNA sequences **(A)**, SNaPshot assay **(B)**, and digested PCR fragments with restriction enzymes as indicated **(C) **revealed the mutations at D178N (GAC→AAC), E200K (GAG→AAG)-129MV (ATG→GTG), and M232R (ATG→AGG). **(A) **Arrow heads indicate the mutated nucleotide peaks. **(B) **The x and y axes represented the size of bases and the fluorescence intensities, respectively. The color of each peak indicates the identity of the ddNTPs in the primer extension reaction, emitting specific fluorescences according to ddNTP; Green/A, Black/C, Blue/G, and Red/T. The orange peaks represented the internal size standard. The arrows labeled "129" or "200" indicate SNaPshot products resulting from the extensions of the S129 and S200 primers, respectively.

After amplifying and sequencing *PRNP *from genomic DNA of probable CJD patients in Korea, three *PRNP *mutations were found (Figure 2A). In Case 1, a single base substitution at codon 178, from GAC to AAC, was found for the amino acid mutation from Asp to Asn. In Case 2, two point mutations were discovered: GAG to AAG (Glu to Lys) at codon 200, and ATG to GTG (Met to Val) at codon 129. Case 3 had one point mutation at codon 232(ATG to AGG), resulting Met replacement with Arg. As shown in the electrophotograms (Figure 2A), two overlapping peaks for the heterozygotes were observed in Cases 1, 2, and 3 patients, in contrast to the homozygote peaks for the healthy control. To confirm the DNA sequencing results in Figure 2A, a SNaPshot assay was performed with the PCR products of *PRNP *from Cases 1, 2, and 3 (Figure 2B). The PCR products from patients with mutations confirmed two peaks, in comparison to a single peak from the healthy control. Two black and red peaks at codon 178 represented heterozygotes of Asp and Asn, while only one peak appeared in homozygotes. Four peaks were apparent in Case 2 since two codons in *PRNP*, at 200 and 129, could be translated to heterozygotes in probable CJD patients. The two peaks for codon 129 (indicated with arrows) were heterozygotes for Met and Val, and the second set of two peaks was from the heterozygotes for Glu and Lys. The heterozygotes at codon 232 for Case 3 represented two peaks of Met and Arg (Figure 2B). Both Case 1 and Case 3 showed Met/Met in codon 129, and the other *PRNP *mutations and polymorphisms were not found in Cases 1, 2, and 3 (data not shown).

### RFLP

The results from RFLP with a different batch of *PRNP *PCR products from DNA sequencing and SNaPshot assay verified the three *PRNP *mutations. In Case 1, the mutation at codon 178 abolished digestion with Tth111 I. Conversion of one allele to Asn presented a band of 680 bp, while RFLP from the healthy control showed two bands of 223 and 456 bp, respectively (Figure 2C). PCR products from the healthy control yielded bands of 75, 296, and 309 bp after Nsp I digestion, while mutation at codon 129 in Case 2 had an additional band of 384 bp. The bands of 296 and 309 bp were not differentiated due to limitation in the separation of fragments with similar size in the DNA gel. In addition, after incubating the PCR products with BsmA I, the digestion site disappeared for the mutation at codon 200 fragment in Case 2. BsmA I digestion of the wild type PCR products at codon 200 yielded two fragments (167 and 513 bp, respectively), while the codon 200 variant yielded fragments of 167, 513, and 680 bp (Figure 2C). In Case 3, Nla III digested the wild type PCR products into two fragments, of 129 and 83 bp, respectively. The AGG substitution at codon 232 abolished the Nla III digestion site in the PCR products, yielding a full-sized 212 bp fragment (Figure 2C). The result of RFLP was tantamount to that of DNA sequencing and SNaPshot.

### MRI and EEG

**Figure 3:**
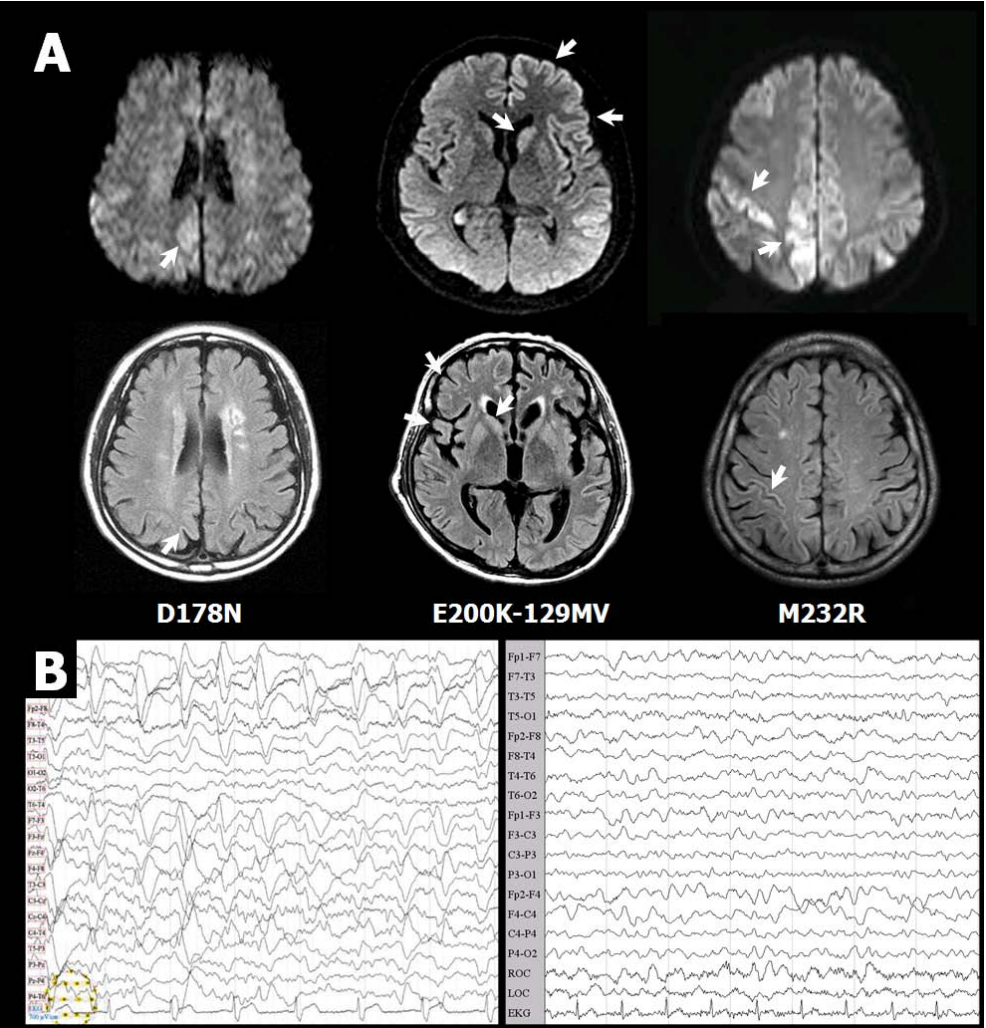
**CJD profiles of MRI and EEG from probable CJD patients**. **(A) **Brain MRIs of Cases 1, 2, and 3 with *PRNP *polymorphisms. The two images at the top and bottom left are of Case 1 with the D178N-129M mutation; the two images at center are of Case 2 with the E200K-129MV mutation; and the two images at top and bottom right are of Case 3 with M232R mutation. The top three are DWI images and the bottom three are T2-FLAIR images. The white arrow indicates a lesion with a high signal. **(B) **EEG of Case 2 (left panel) and Case 3 (right panel). The EEG of Case 1 was normal (data not shown).

In Case 1, lacunar infarctions appeared in the pons, left thalamus, and both putamen and cerebral white matter. Diffuse cerebral atrophy was more prominent in the frontoparietal lobes. Some high signal intensities were observed along gyri of both parietal and occipital lobes in diffusion and fluid-attenuated inversion-recovery (FLAIR) images (Figure 3A). For EEG analysis, 32 channel digital EEG monitoring was performed with the patient in an awake state. The posterior dominant rhythm was less well regulated with 8~9 Hz activity, which was reactive to eye opening. There was no definite interictal epileptiform discharge or focal slowing, and the patient was diagnosed as normal EEG (data not shown).

In Case 2, the diffusion-weighted MRI (DWI) showed higher signal intensities in the left side than the right side of basal ganglia or the cerebral cortex (Figure 3A). After 50 days, the second MRI revealed higher signal intensities in the right side of the cerebral cortex than the left, indicating damage to the right hemisphere of the brain. The follow-up FLAIR images indicated atrophy in the right frontal lobes and caudate nucleus (Figure 3A). Frequent generalized rhythmical (0.5~1.5 Hz) slow activities were observed, which consisted of sharp or spike and slow waves with very high amplitude, more from the left cerebral hemisphere (Figure 3B).

In Case 3, DWI showed high signal intensities mainly from the cortex of the parieto- occipital and temporal lobes, and FLAIR images showed high signal intensity lesion from the cortex of temporal lobe (Figure 3A). The EEG results revealed slow waves ranging from diffuse theta to delta (Figure 3B).

## Discussion and Conclusion

The genotype of the patients seemed to play an important role in the pathogenesis of CJD. The D178N mutation abolished the salt bridge between the conserved residues Asp-178-Arg-164, suggesting that the mutant protein had reduced thermodynamic stability [21]. Moreover, Asp-178 was in the vicinity of the disulfide bridge (S-S), and mutation in this region could potentially interfere with the formation of disulfide bridge, and potentially cause protein aggregation [22]. The E200K mutation indicated a significant reduction in glycosylation in comparison to the wild-type, which could affect to the conformational changes in prion protein [23]. An analogous reduction in glycosylation was reported in fCJD patients with the E200K mutation [24]. Codon 232 at the carboxy-terminal region in the PrP was implicated in post-translational modification by the glycosylphosphatidylinositol anchor during protein maturation [25]. It seemed that arginine substitution for methionine at codon 232 prohibited the cleavage during the glycosylphosphatidylinositol anchor biosynthetic process, resulting in conformational changes in mutant PrP [12].

The D178N mutation with 129MM was the genotype for FFI and sCJD with 129MV or 129VV [26]. Japanese CJD patients with the D178N mutation with 129MM did not have clinical insomnia [27], whereas Chinese patients with same genotype were diagnosed with typical FFI [28]. Furthermore, D178N didn't show only FFI phenotype all the time in case of not only Asia but also Europe, but sometimes showed CJD phenotype which shows phenotypic variability [29]. The Korean patient with the D178N mutation with 129MM had a similar phenotype to the Japanese CJD patients, so we suggest that other potential factors may be related to the clinical features. The symptoms of the patient with the M232R mutation was similar to those of Japanese CJD patients with the M232R mutation [12]. The patient is considered a slow type of patients with M232R, due to not showing akinetic mutism or PSWC [30]. Despite differences in ethnicity, the clinical and pathological outcomes were similar to the respective mutations around the world. Autopsies were not performed in all cases, since the Korean tended to avoid in performing autopsies due to the traditional Confucianism culture. Therefore, we need a development substitute diagnostic method and we consider that a clinical view of DWI and FLAIR imaging may be useful in the early premortem diagnosis of CJD.

The D178N-129M, E200K-129MV, and M232R mutations, which were previously reported from other countries, were identified for the first time among probable CJD patients in Korea. Diverse *PRNP *polymorphisms in the Korean population will be investigated in the future. *PRNP *polymorphisms among Korean, Chinese, and Japanese populations will provide insights into the associations between polymorphisms, CJD susceptibilities, and their clinical phenotypic variations, especially when 129MM codon has been reported to be a dominant determinant in more than 90% of populations.

## Competing interests

The authors declare that they have no competing interests.

## Authors' contributions

BY conducted the Western blotting, genetic analysis, RFLP and drafted the manuscript. SY(2^nd^), SY(3^rd^) and SS designed, drafted and reviewed the manuscript. SY, SE, YJ, SH and SJ performed the clinical observations and provided MRI and EEG pictures. CK and JS supported the process of examination and reviewed the manuscript. YR overviewed the work. All authors read and approved the final manuscript.

## Pre-publication history

The pre-publication history for this paper can be accessed here:

http://www.biomedcentral.com/1471-2334/9/132/prepub
